# Physicochemical Properties of Sago Ozone Oxidation: The Effect of Reaction Time, Acidity, and Concentration of Starch

**DOI:** 10.3390/foods10061309

**Published:** 2021-06-07

**Authors:** Siswo Sumardiono, Bakti Jos, Isti Pudjihastuti, Arvin M. Yafiz, Megaria Rachmasari, Heri Cahyono

**Affiliations:** 1 Department of Chemical Engineering, Faculty of Engineering, Universitas Diponegoro, Semarang 50239, Indonesia; bakti.jos@che.undip.ac.id (B.J.); arvinmuhammadyafiz@gmail.com (A.M.Y.); rachmasarimegaria@gmail.com (M.R.); hericahyono@che.undip.ac.id (H.C.); 2 Department of Industrial Chemical Engineering, Vocational School, Universitas Diponegoro, Semarang 50239, Indonesia; istipudjihastuti@gmail.com

**Keywords:** starch, oxidation, physicochemical properties

## Abstract

The disadvantageous properties of sago starch has limited its application in food and industrial processes. The properties of sago starch can be improved by changing its physicochemical and rheological characteristics. This study examined the influence of reaction time, acidity, and starch concentration on the oxidation of sago starch with ozone, a strong oxidant. Swelling, solubility, carbonyl, carboxyl, granule morphology, thermal profile, and functional groups are comprehensively observed parameters. With starch concentrations of 10–30% (*v*/*w*) and more prolonged oxidation, sago starch was most soluble at pH 10. The swelling power decreased with a longer reaction time, reaching the lowest pH 10. In contrast, the carbonyl and carboxyl content exhibited the same pattern as solubility. A more alkaline environment tended to create modified starch with more favorable properties. Over time, oxidation shows more significant characteristics, indicating a superb product of this reaction. At the starch concentration of 20%, modified sago starch with the most favorable properties was created. When compared to modified starch, native starch is generally shaped in a more oval and irregular manner. Additionally, native starch and modified starch had similar spectral patterns and identical X-ray diffraction patterns. Meanwhile, oxidized starch had different gelatinization and retrogradation temperatures to those of the native starch.

## 1. Introduction

Starch, which is mainly composed of carbohydrates, is an essential daily nutrient source. Starch can be produced from various plant parts, such as the seeds of corn, rice, wheat, tubers of cassava, yams, and potato, and the stem of sago palm [1]. Starch plays an essential role in the food industry, such as in the production of candy, glucose, dextrose, and fructose. Besides, it is widely used in other industries, such as textile, paper, glue, and sludge drilling [2,3,4,5,6]. There are two kinds of starch that are produced in agriculture: native and modified [7]. Various starch modifications have been carried out using various starch sources, one of which can be developed from sago.

The sago palm (*Metroxylone sagu* Rottb) grows well in Southeast Asia’s tropical rain forests between 100 northern and southern latitudes [8,9]. Sago starch contains 73% amylopectin as the branched polymer and 27% amylose (the linear polymer) [10,11]. While native sago starch can be a valuable source of foodstuffs and industrial raw materials, its use of native sago starch is still limited due to the disadvantageous properties of the raw sago paste [11]. For example, raw sago paste becomes a chewy paste when heated, is difficult to dissolve in cold water, opaque, spoils quickly during storage, and it lacks emulsifying properties. Consequently, industrial applications of sago starch are limited [12,13], and most have not been sufficiently utilized. Native sago starch is also reactive, because it contains free hydroxyl groups on carbon 2, 3, and 6 of its glucose molecules [13,14]. Thus, raw sago paste affects the properties of a food product. Modification becomes a solution for overcoming the lack in native starch.

Similar to other native starches, the quality and properties of sago starch can be improved by changing sago starch’s physicochemical and rheological characteristics [15]. Modification efforts aim to reduce the weakness of native starch to increase its application on various products [13,16]. Industries require modified sago starch to have specific properties, such as viscosity at high and low temperatures, increased resistance to mechanical treatment, and resistance to viscous force under acidic and high-temperature conditions [17]. It has been found that the process of heating and shearing reduces sago starch’s high viscosity; sago starch is further broken down under acidic conditions. Some methods have recently been developed to modify starch with acid, enzyme, oxidation, or crossing bond [7,11,18,19].

Starch molecules have many hydroxyl groups (C–OH). During oxidation, the hydroxyl groups are substituted with carboxyl (HO–C=O) and carbonyl (C=O) groups; an increase in the content of carboxyl and carbonyl, causes the degree of substitution to increase [20]. The carbonyl and carboxyl values depend on various factors, such as starch type, oxidizing type, starch concentration, reaction time, temperature, and pH [21]. The carbonyl group (C=O) is a carbon atom that is bonded to two atoms. Aldehydes and ketones are carbonyl compounds that only have hydrogen, an alkyl, or an aryl group attached to the carbonyl carbon atom. Carbonyl bonds affect carbonyl bonds’ stability and reactivity [22]. The presence of an oxidizing agent causes a change in the physicochemical properties of starch [21].

Ozone, a strong oxidizing agent, is widely used in water and wastewater treatment, because it can remove odor, color, taste, and soluble particles in water or suspensions. It has many applications in food processing [7,23,24,25,26]. Several previous studies confirmed that ozone could change the physicochemical, structural, and rheological starch characteristics [23,24]. Ozone is more soluble in water than oxygen, so that strengthening ozone can be applied in suspension in water [27]. This solubility will increase the likelihood of contact between the ozone particles and starch in the oxidation reaction [27]. The nature of ozone (O_3_) naturally changes into oxygen (O_2_) quickly. It will not leave any residue in the product [28]. Ozone presents several advantages as an agent for modification, which makes it very suitable for modifying sago starch.

Chan et al. (2009) have successfully modified sago starch with ozone; however, they have not comprehensively studied the morphological properties, functional groups, and thermal properties of the modified sago starch [29]. Several previous studies have reported a positive effect of ozone intervention on the physicochemical properties of sago starch. However, no information is gained on the thermal effects of ozone oxidation treatment, as well as the morphological and functional group properties of the resultant modified sago starch. This study aims to provide a more comprehensive examination of the application of ozone gas as an oxidant, the effect of reaction time, acidity, and starch concentration on the oxidation reaction with ozone, and the physicochemical properties, granule morphology, thermal profile, and functional groups of the non-residual modified sago starch.

## 2. Materials and Methods

### 2.1. Materials

Alfurqan Tribinatama company supplied sago starch “Engke” (*Metroxylon sagu*). Ozone was produced with an Ozonator (Aquasuper model LZH-103A) Moro Jaya Teknik, Jakarta, Indonesia with an oxygen supply (99.60–99.9%) from Samator Aneka Gas Industri. Merck provided NaOH, potassium iodide, amylum, HCl, AgNO_3_, NH_2_OH, Cl, and phenolphthalein indicator.

### 2.2. Sago Starch Modification

A total of 100 gr dry starch was dissolved in water at 10%, 20%, and 30% and then added into a stirring tank reactor. In this study, the ozone rate was 1.5 L/min. with a pH of 4, 7, and 10, while the reaction time of ozonation was 5, 10, 15, 20, and 25 min. Adding the HCL or NaOH solution was he acidity of the solution was adjusted by a.

The samples were filtered using a vacuum pump; then, the modified sago starch was dried for several days at room temperature of 28 °C and an atmospheric pressure of 1 atm. The dried modified sago starch was analyzed using swelling power, solubility, carbonyl, and carboxyl chain tests, as well as scanning electron microscope (SEM), X-ray diffraction (XRD), Transform Infra-Red (FTIR), and differential scanning calorimetry (DSC) analyses.

### 2.3. Evaluation of Modified Sago Starch

#### 2.3.1. Swelling Power Test

The swelling power of modified sago starch was determined using the method outlined by Chan et al. (2009) [29]. One-gram samples were dispersed in 10 mL water. The solution was heated in a 60 °C water bath for 30 min. Subsequently, the supernatant and the sago starch paste were separated by centrifuging at 2500 rpm for 15 min. The swelling power was calculated using the following equation:

(1) $$\mathrm{Swelling}\;\mathrm{power}\;\left(\%\right)=\frac{\mathrm{paste}\;\mathrm{weight}}{\mathrm{weight}\;\mathrm{of}\;\mathrm{dry}\;\mathrm{sample}}$$

#### 2.3.2. Solubility of Sago Starch in Water

The solubility of modified sago starch was determined using the method presented by Chan et al. (2009) [29]. One-gram samples were dispersed in 10 mL aquadest. The solution was heated in a 60 °C water bath for 30 min. and then centrifuged at 3000 rpm for 20 min. to separate the paste from the supernatant. A drying cup was filled with 10 mL of the supernatant, which was then into the oven at 105 °C. The dry residue’s constant weight was recorded, and sago starch’s solubility was calculated, as follows.

(2) $$\mathrm{Solubility}\;\left(\%\right)=\frac{\mathrm{weight}\;\mathrm{of}\;\mathrm{dry}\;\mathrm{residue}}{\mathrm{volume}\;\mathrm{supernatant}}$$

#### 2.3.3. Carboxyl Test

The modified sago starch’s carboxyl was determined using the method that was developed by [30]. Three-gram samples were dispersed in 25 mL of 0.1 N HCl, stirred for 30 min., and then filtered. The slurry was then washed until there was no longer any Cl^−^ content. The filtrate was tested for Cl^−^ content using AgNO_3_. If there were Cl^−^ in the slurry, its color would be turbid. Subsequently, 300 mL of aquadest was added to the slurry that was free of Cl^−^. The solution was heated in boiling water until gelatin formed; heating was then continued for another 15 min. The hot solution was titrated with 0.1 N NaOH using phenolphthalein as an indicator. The carboxyl chain test was repeated using native sago starch. The percentage of carboxyl was calculated, as follows.

(3) $$\mathrm{Carboxyl}\;\left(\%\right)=\frac{V.\;\mathrm{NaOH}\;\mathrm{modified}\;\mathrm{starch}-V.\;\mathrm{NaOH}\;\mathrm{native}\;\mathrm{starch}}{\mathrm{sample}\;\mathrm{weight}\;\left(g.\mathrm{dry}\;\mathrm{basis}\right)}\times N\;\mathrm{NaOH}\times 0.045\times 100$$

#### 2.3.4. Carbonyl Test

The carbonyl of the modified sago starch was determined using the method outlined by Sangseethong et al. [30]. Four-gram samples were dispersed in 100 mL of aquadest, heated for 20 min. until gelatin was formed, and then cooled to 40 °C. The solution’s pH was adjusted to 3.2 by adding 0.1 N HCl. Twenty-five g of hydroxylamine hydrochloride was dissolved into 100 mL 0.5 N NaOH and then brought up to 500 mL by adding aquadest. Additionally, 15 mL of hydroxylamine was added to each sample solution. The solution was stirred in the tube and kept in a 40 °C water bath for four hours before titrating to pH 3.2 with 0.1 N HCl. A clean sample with the only hydroxylamine was measured in the same step. The hydroxylamine reagent was prepared by dissolving 25 g of hydroxylamine hydrochloride in 100 mL of 0.5 N NaOH. The final volume was then adjusted to 500 mL with distilled water.

(4) $$\mathrm{Carbonyl}\;\left(\%\right)=\frac{\left[\left(V.\;\mathrm{sample}-V.\mathrm{native}\;\mathrm{starch}\right)\times N\;\mathrm{HCl}\times 100\right]}{\mathrm{sample}\;\mathrm{weight}\;\left(g.\;\mathrm{dry}\;\mathrm{basis}\right)}\times 0.028$$

#### 2.3.5. Morphology Analysis

An analytical scanning electron microscope (SEM-EDX JEOL JSM-6510LA, JEOL Ltd., Tokyo, Japan) with 2000× magnifications was used to observe the granule surfaces of native and modified sago starch. SEM is a microscopic electron method used to analyze a sample’s morphology. It produces a sample image by scanning the sample with focused light from an absorption electron that produces an electron beam at an accelerated voltage of 2–30 kV [10,31].

#### 2.3.6. Functional Group Analysis

The functional group of modified sago starch was observed using a Fourier Transform Infra-Red (FTIR) Shimadzu (type IRprestige 21, Shimadzu Corporation, Kyoto, Japan).

#### 2.3.7. Thermal Profile

A differential scanning calorimeter was used to determine the temperature profiles of native starch and modified sago starch (Type DSC-60, Shimadzu Corporation, Kyoto, Japan). Scanning was carried out at a temperature range of 30–200 °C with a scanning speed of 10 °C/minute. An empty pan was used as a reference, and the data were analyzed by TA-60WS software (Shimadzu Corporation, Kyoto, Japan).

### 2.4. Statistical Analysis

The analyses were repeated twice for each sample. The data obtained are shown as the mean value ± standard deviation (Mean ± SD). The data were analyzed using One-way analysis ANOVA. Subsequently, we conducted a post-hoc test with Duncan test at intervals of 95% confidence for further analysis of each of these variables.

## 3. Results

### 3.1. Effect of pH, Time of Reaction, and Starch Concentration on Carbonyl Group Content

**Figure 1:**
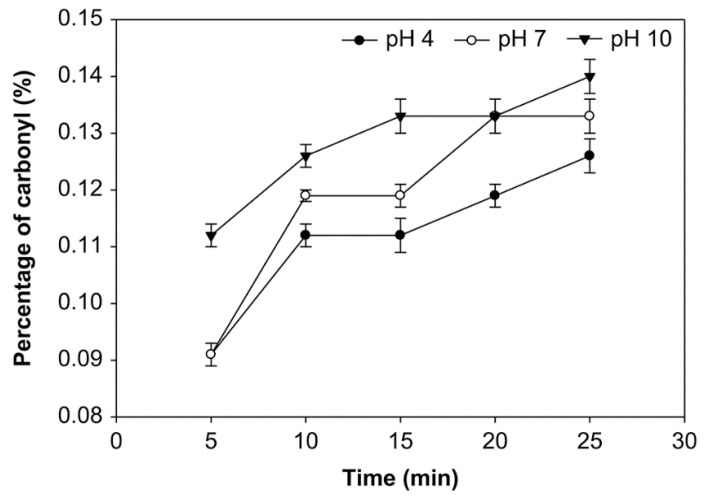
The effect of time on carbonyl group content at various pH with the starch concentration of 10% (*v*/*w*).

**Figure 2:**
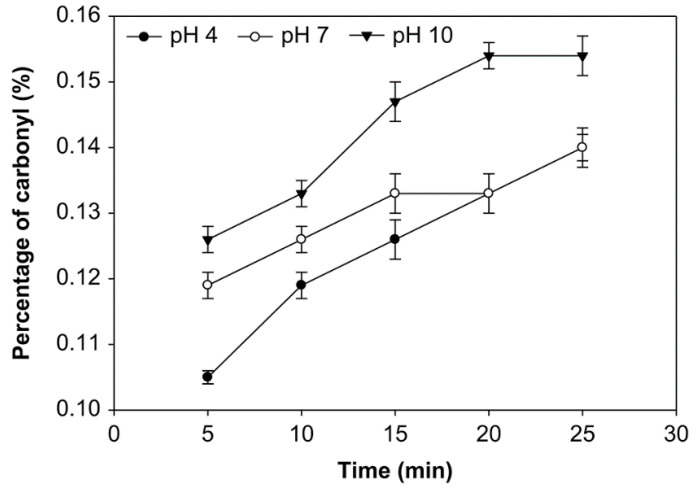
The effect of time on carbonyl group content at various pH and the starch concentration of 20% (*v*/*w*).

**Figure 3:**
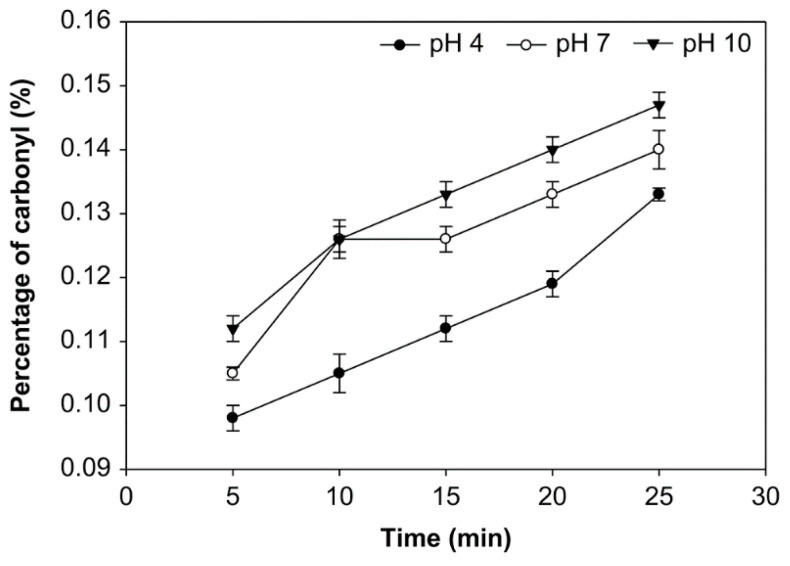
The effect of time on carbonyl group content at various pH with the starch concentration of 30% (*v*/*w*).

The carbonyl group (DSC=O) content is expressed in the number of moles of carbonyl per mole of anhydroglucose unit [32]. The effects of pH, reaction time, and starch concentration on the carbonyl group content were examined (Figure 1, Figure 2 and Figure 3). The number of carbonyl groups substituted for the modified starch increased at pH 4, 7, and 10 and the starch concentration of 10%, 20% and 30% (*v*/*w*), and with longer oxidation time. On the other hand, at starch concentrations of 10%, 20%, and 30% (*v*/*w*), the carbonyl percentage at pH 10 was higher than pH 7 and pH 4. Additionally, the average number of carbonyl groups obtained at starch concentrations of 10%, 20%, and 30% was not significantly different (*p* > 0.05), ranging from 0.091% to 0.154%. The modified starch had the highest carbonyl percentage at 0.154% with a starch concentration of 20%, reaction time of 25 min. and pH 10.

### 3.2. Effect of pH, Time of Reaction, and Starch Concentration on Carboxyl Group Content

**Figure 4:**
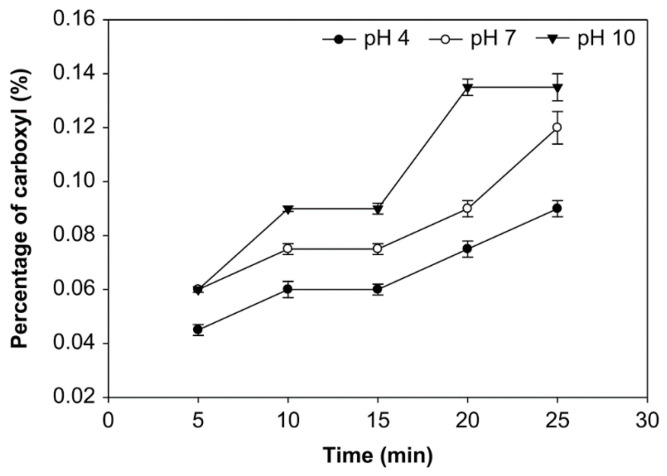
The effect of time on carboxyl group content at various pH with the starch concentration of 10% (*v*/*w*).

**Figure 5:**
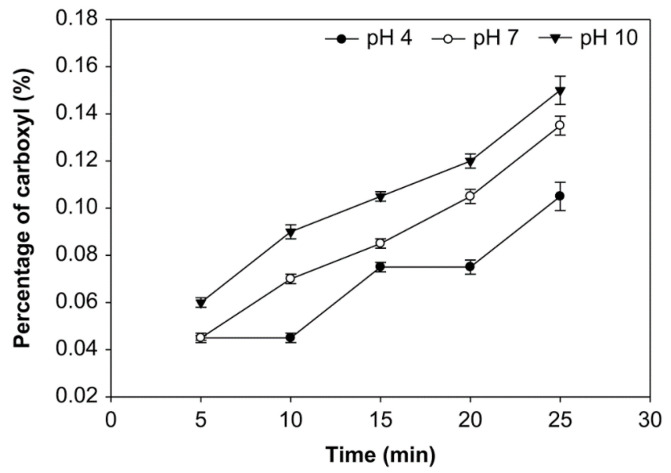
The effect of time on carboxyl group content at various pH with the starch concentration of 20% (*v*/*w*).

**Figure 6:**
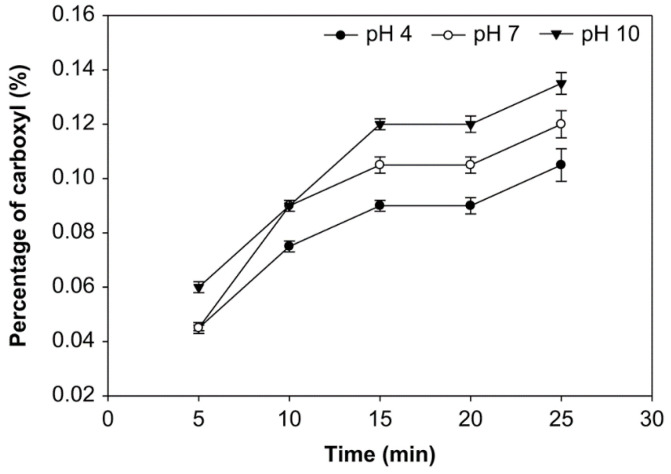
The effect of time on carboxyl group content at various pH with the starch concentration of 30% (*v*/*w*).

The carboxyl group content of the modified starch was obtained titrimetrically and expressed in the number of carboxyl moles per mole of anhydroglucose units [32]. The outcome of reaction time, pH, and starch concentration on the carboxyl group was examined (Figure 4, Figure 5 and Figure 6). More carboxyl groups were generated with a longer oxidation time at pH 4, 7, and 10 and starch concentrations of 10%, 20% and 30% (*v*/*w*). Additionally, at starch concentrations of 10%, 20%, and 30%, the carboxyl group content at pH 10 was higher than at pH 7 and 4. Additionally, the average number of carboxyl groups obtained at various starch concentrations was not significantly different (*p* > 0.05), ranging from 0.045% to 0.150%. At the starch concentration of 20%, the carboxyl group content reached the highest value at 0.150% at 25 min. and pH solution 10.

### 3.3. Effect of pH, Time of Reaction, and Starch Concentration on Solubility of Starch

**Figure 7:**
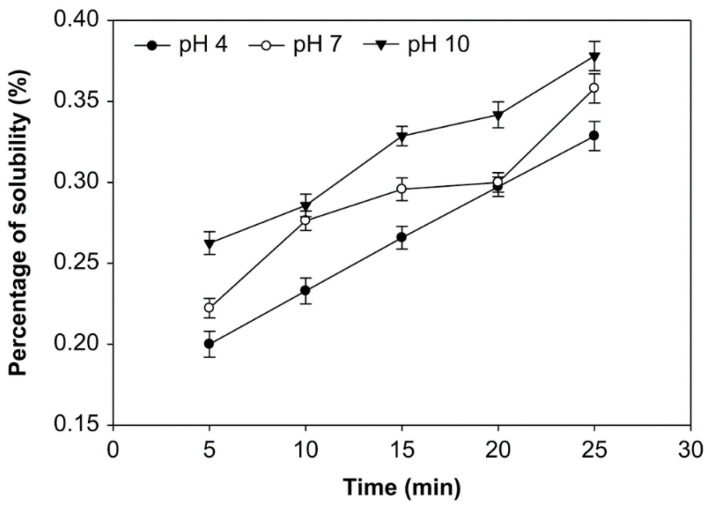
The effect of time on solubility at various pH with the starch concentration of 10% (*v*/*w*).

**Figure 8:**
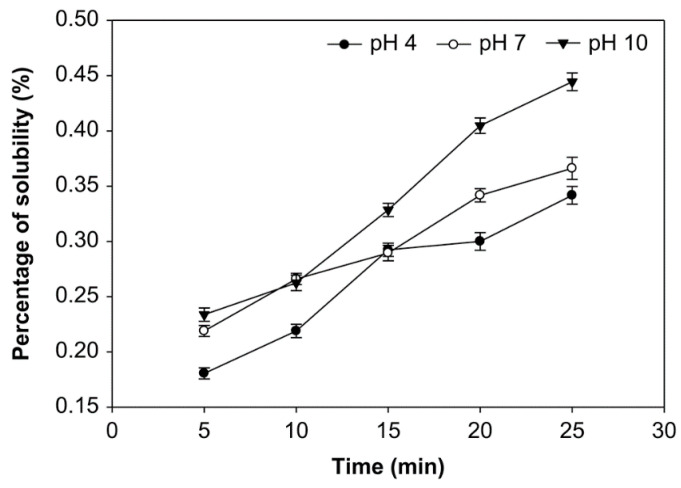
The effect of time on solubility at various pH with the starch concentration of 20% (*v*/*w*).

**Figure 9:**
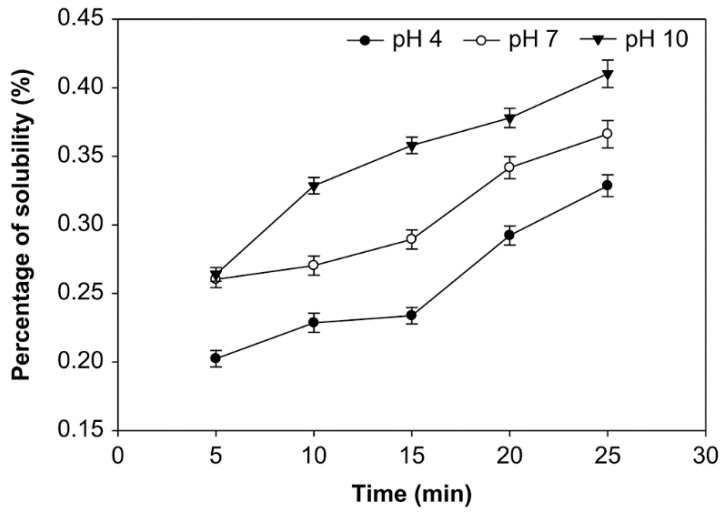
The effect of time on solubility at various pH with the starch concentration of 30% (*v*/*w*).

Solubility is the number of starch molecules that are dissolved at a specific temperature; it is expressed as grams of dry residual weight per 100 g of dry sample weight [21,29]. The starches’ solubility at various pH, starch concentrations, and ozonation time were examined (Figure 7, Figure 8 and Figure 9). The modification of native sago starch by oxidation using ozone as an oxidant produced a modified starch with higher solubility, which changed the starch’s rheological and psychochemical properties.

### 3.4. Effect of pH, Time of Reaction, and Starch Concentration on the Swelling Power of Starch

**Figure 10:**
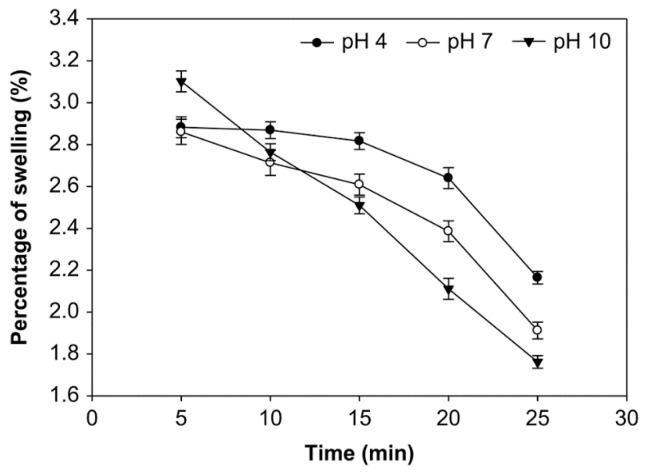
The effect of time on swelling power at various pH with the starch concentration of 10% (*v*/*w*).

**Figure 11:**
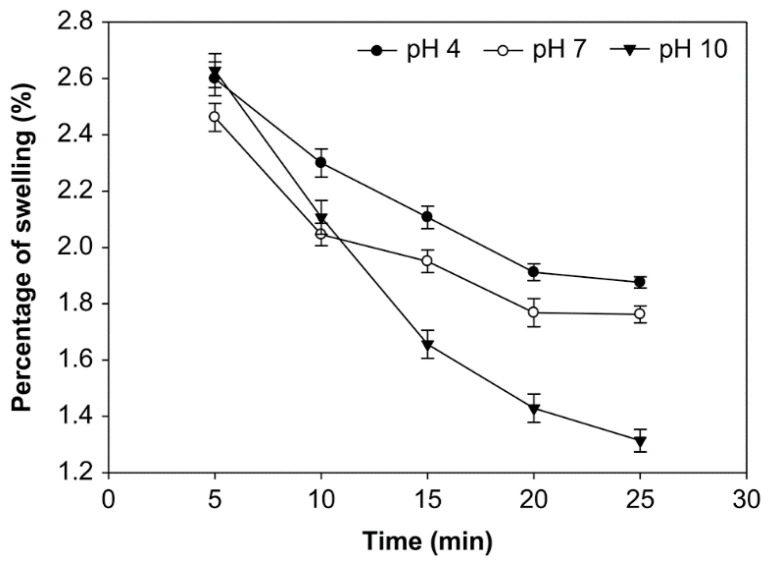
The effect of time on swelling power at various pH with the starch concentration of 20% (*v*/*w*).

**Figure 12:**
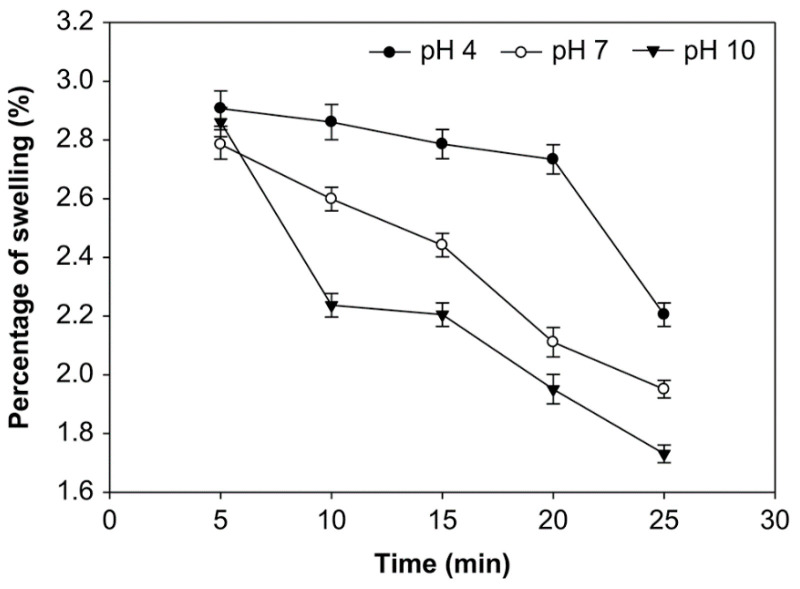
The effect of time on swelling power at various pH with the starch concentration of 30% (*v*/*w*).

The effect of reaction time, pH, and starch concentration on the swelling power was examined (Figure 10, Figure 11 and Figure 12). A longer oxidation time decreased the swelling power at pH 4, 7, and 10 and starch concentrations of 10%, 20%, and 30% (*v*/*w*). On the other hand, at starch concentrations of 10%, 20%, and 30%, the swelling power at pH 10 was smaller than pH 7 and 4. The average swelling power at starch concentrations 10%, 20%, and 30% was not significantly different (*p* > 0.05), ranging from 1.314% to 3.102%. At the starch concentration of 20%, oxidation time of 25 min., and pH 10, the swelling power had the smaller value at 1.314%. The swelling power of native starch is higher, reaching 3.150%. The swelling power of starch has decreased after oxidation due to reduced amylose content [33]. Amylopectin increases swelling and attaches starch granules, while amylose and fat inhibit swelling [34].

### 3.5. Effect of pH and Starch Concentration on Starch Granule

**Figure 13:**
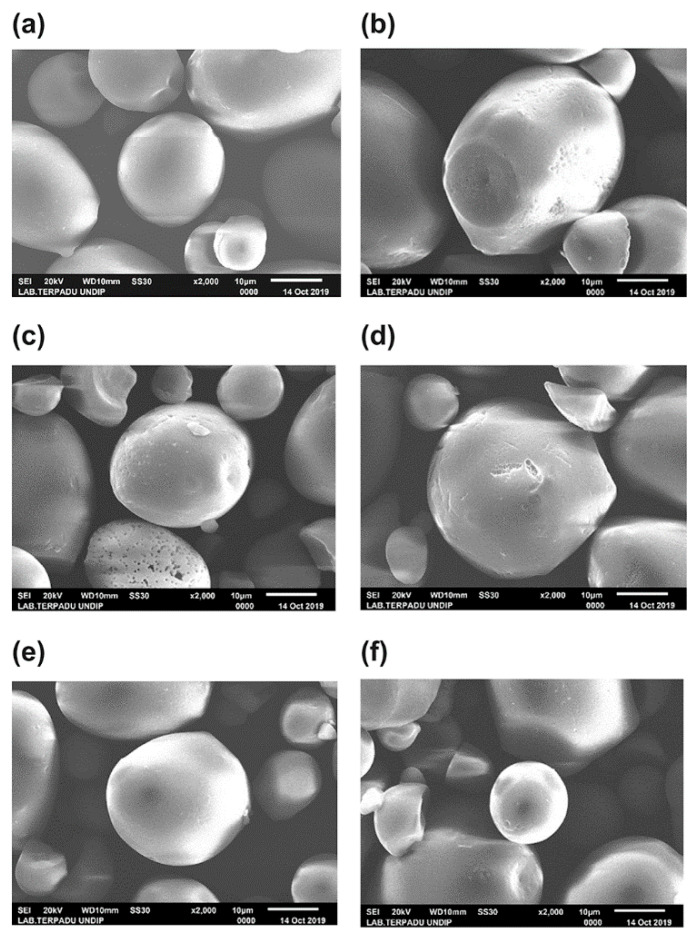
Scanning electron micrographs of sago starches. (**a**) Native starch. (**b**) Modified starch with ozone-oxidized treatment at pH 4 and a starch concentration of 30%. (**c**) Modified starch at pH 10% and 20% starch. (**d**) Modified starch at pH 7 and 20% starch. (**e**) Modified starch at pH 4 and 20% starch. (**f**) Modified starch at pH 4 and 10% starch. Magnification: 2000×.

SEM was used to compare the morphology of the native and modified starch granules at various pH values and starch concentrations with an oxidation time of 25 min (Figure 13) in order to detect any change after oxidation. Most native sago starch granules are smooth and oval-shaped, but some are irregularly shaped (Figure 13a). Meanwhile, oxidized starch granules exhibit a smooth surface with few pores and defects [11,35]. Similar findings have been reported in other studies [36,37]. Some granules have cut ends, but most of them are visible. The granule surface of the native starch is smooth and relatively free of imperfections (Figure 13a); this observation is consistent with observations of other native starch granules [38].

### 3.6. Effect of pH and Starch Concentration on the Functional Groups of Starch

**Figure 14:**
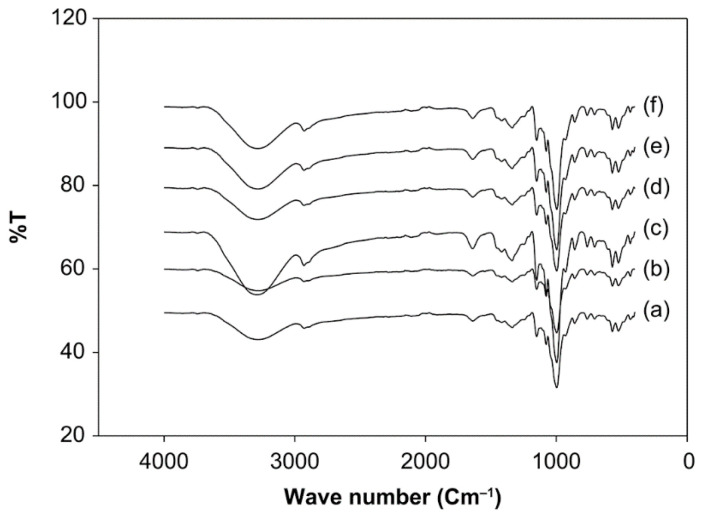
The FTIR spectra of native and modified sago starches. (**a**) Native starch. (**b**) Modified starch with ozone-oxidized treatment at pH 4 and the starch concentration 30%. (**c**) Modified starch at pH 10% and 20% starch. (**d**) Modified starch at pH 7 and 20% starch. (**e**) Modified starch at pH 4 and 20% starch. (**f**) Modified starch at pH 4 and 10% starch.

FTIR analysis was used to compare the absorption at specific wavelengths between the native starch and modified starch (Figure 14). The spectral patterns of all the samples were similar. In the present study, sago starch exhibited more than 10 peaks in 4000–400 cm^−1^. The bands at 3000–3750, 2700–3000, and 1600–1800 cm^−1^ were assigned to stretch O–H, stretch C–H, and bend water, respectively [39]. The bending vibrations of CH2 or CH3 from protein or lipid side chains appeared in the FTIR band of 1200–1475 cm^−1^ [40]. The anhydrous C=O strain of the glucose ring appears in the 990 and 1030 cm^−1^ bands. Additionally, the bonding to the carbonyl group, C=O, displayed absorption in the region of 1680–1750 cm^−1^. The O–H bond is another type of bond that is important for identification; it absorbs at different positions, depending on the environment. This bond is very easily identified as acidic because it produces a wide signal in the area of 2500–3300 cm^−1^ [41,42].

### 3.7. Effect of pH and Concentration of Starch in Water on the Crystallinity of Starch 

Diffraction as a physical phenomenon involves electromagnetic waves at a specific wavelength from a specific metal; thus, elastic reflections can be detected by varying the angle of reflection. The analysis of the materials as the atom plans are placed at comparable distances to the X-ray lengths can be explained as the application of this phenomenon [43]. Here, XRD is used to characterize the composition of native and modified starches by analyzing their respective crystal structure. The positions and numbers of the peaks provide insight into the crystal family, a system for organizing and classifying the crystallinity of substances. Crystals are defined as substances with repeating structures; a crystal is divided into repeating parts or motifs [44]. The crystal structure of material primarily determines its properties and structure; XRD techniques can determine the position of the atoms in a crystal with an accuracy in the order of 10^−4^ nm [45].

**Figure 15:**
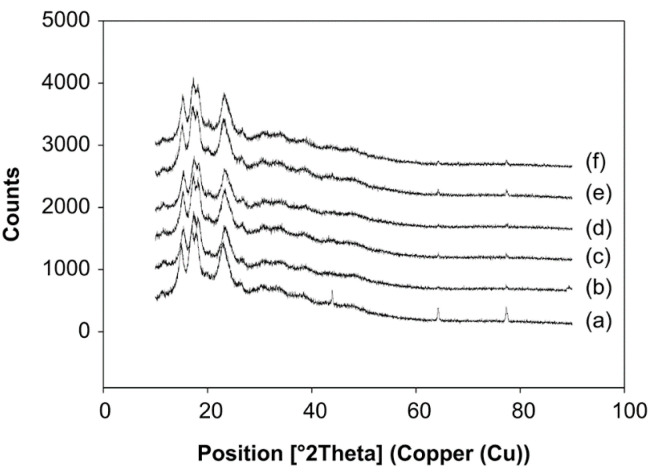
X-ray diffraction pattern of native and modified sago starch. (**a**) Native starch. (**b**) Modified starch with ozone-oxidized treatment at pH 4 and the starch concentration of 10%. (**c**) Modified starch at pH 4 and 20% starch. (**d**) Modified starch at pH 4 and 30% starch. (**e**) Modified starch at pH 7 and 20% starch. (**f**) Modified starch at pH 10% and 20% starch.

The XRD patterns of the native and ozone-oxidized sago starches were compared (Figure 15). The oxidized sago starches displayed XRD patterns that were similar to those of the native starch. The level of crystallinity of the starch is influenced by: (1) the number of crystal regions, which is influenced by the content and the crystal chain; (2) crystal size; (3) the level of interaction between the double helix; and, (4) the orientation of the double helix in the crystal area [31].

### 3.8. Effect of pH and Starch Concentration on the Thermal Properties of the Starch 

Differential scanning calorimetry (DSC) is used to determine starch’s thermal behavior [46]. The thermal profile of oxidized starch was different from that of the native starch, as shown in Table 1. For some variables, i.e., To, Tp, and Tc, under some conditions, the resultant oxidized starch has higher To, Tp, and Tc than native starch, which indicates that ozonation produced higher thermal stability starch.

## 4. Discussion

The carbonyl group content shown in Figure 1, Figure 2 and Figure 3 increased at pH 4, 7, and 10, starch concentrations of 10%, 20%, and 30% (*v*/*w*), and with an increased time of oxidation because more oxidants converted the hydroxyl groups into the carbonyl groups. Hydrogen bonds influence the integrity of the native starch grains. The decreasing of hydrogen bonds between the modified starch molecules occurs due to hydroxyl group replacement. Thus, the carbonyl and carboxyl group formation were considered to cause granular weakening [20]. The carbonyl group contents increase as a function of the ozonation reaction time [24]. Chan et al. observed similar results and concluded that the carbonyl group content increased with increased oxidation [29]. Another effect of modifying ozone starch using different variables and processes shows that the carbonyl group content increases with increasing ozonation [20,47]. 

While at various starch concentrations, the carbonyl group content at pH 10 was higher than that at pH 7 and 4, likely because carbonyl groups formed quickly at pH 7–7.5 [25]. Starch oxidation is the most efficient under ideal conditions at 7.5–9, followed by pH 6.5–9.5 and pH 6–10. It has been found at these pH ranges, a small amount of oxidizing agent is sufficient for producing oxidized starch with excellent properties [48]. Therefore, at pH 4, the carbonyl group substitution was less than at another pH. However, the pH during the reaction was kept below 10.5 because ozone broke down at higher pH (>10). On the other hand, the ozonation of cassava starch in an aqueous solution at dissimilar pH produced starch with significantly different carbonyl contents; a higher carbonyl content was achieved with ozone oxidization at the alkaline pH of 9.5 than the reactions at pH 6.5 and 3.5 [31].

The number of carboxyl groups increased at pH 4, 7, and 10, the starch concentrations of 10%, 20%, and 30% (*v*/*w*), and longer oxidation time due to the increased change of hydroxyl groups into carboxyl groups, as shown in Figure 4, Figure 5 and Figure 6. The increase of carboxyl group contents was due to the free hydroxyl group’s oxidation at C2, C3, and C6, thus degrading starch and breaking down the amylose chain. The oxidation of hydroxyl groups to carbonyl and carboxyl groups results in the depolymerization of starch molecules by the cleavage (1 → 4) -α-D and (1 → 6) -α-D glycosidic bond occurring during the oxidation of starch. In the case of ozone reaction, the depolymerization of starch becomes more substantial, resulting in increased oxidation of the hydroxyl groups to carbonyl and carboxyl [48].

The carboxyl group content increases with ozonation time [29,47]. Another study examined ozone-assisted of starch modification using different processes and variables; this study also reported that the increase in ozonation elevated the carboxyl group contents [20].

Oxidation will change the hydroxyl group in starch to the carbonyl group. Oxidation will further transform the carbonyl group into carboxyl groups, which breaks the starch’s glycosidic bonds [49]. The carboxyl group consists of a hydroxyl group and carbon that is bonded to oxygen. The carboxyl group’s polarity causes a compound to participate in hydrogen bonding and other important reactions. Thus, the presence of carboxyl groups will affect starch’s physical and chemical properties due to the weakening of the starch’s granular structure after oxidation [29].

Here, at various starch concentrations of 10%, 20%, and 30% (*v*/*w*), the carboxyl group content at pH 10 was higher than that at pH 7 and 4, because carboxyl groups are easily formed at a pH over 8.5 [25]. At a pH in this range, a particularly small number of oxidizing agents will be sufficient for producing oxidized starch with excellent properties [50]. Therefore, at pH 4, the carboxyl group content was smaller than at another pH. However, the ozone decomposes at pH over 10.5 or below 1, so the starch’s hydrolysis is optimal between these pH values. Meanwhile, the carboxyl group content was used to evaluate the ozone’s ability to oxidize cassava starch in aqueous solutions at different pH values. Klein et al. found that the oxidized ozone’s carboxyl content was higher in alkaline conditions, at pH 9.5, than in the acidic conditions of pH 6.5 and 3.5 [31].

The ozonation time, pH, and starch concentrations all appeared to affect the modified starch’s solubility (Figure 7, Figure 8 and Figure 9). The solubility was increased at pH 4, 7, and 10, with starch concentrations of 10%, 20%, and 30% (*v*/*w*) and longer oxidation time. On the other hand, at starch concentrations of 10%, 20%, and 30%, solubility at pH 10 was higher than at pH 7 and 4. However, the average solubility at various starch concentrations was not significantly different (*p* > 0.05), ranging from 0.181% to 0.444%. At the starch concentration of 20%, oxidation time of 25 min., and pH 10, the solubility was highest, at 0.444%. In contrast, the solubility of the native starch was much lower, at 0.138%.

The solubility of modified sago starch is higher than native starch. The duration of starch oxidation increases starch solubility due to depolymerization and the weakening of the structure of the starch granule. The solubility of oxidized sago starch increased with an increasing moles number of oxidizing agents, but not significantly (*p* > 0.05) [20,29]. The starch granule’s weakening is due to carbonyl and carboxyl groups [23]. Thus, increasing the number of carboxyl and carbonyl groups will enhance the solubility of starch. For example, the solubility of oxidized potato starch increases with an increased oxidation time [51].

Regarding the effect of pH on solubility, at pH 10, the modified starch had higher solubility than at pH 7 and 4, because, at pH 10, the number of carboxyl and carbonyl groups generated was higher than at pH 7 and 4. The increase in carboxyl and carbonyl groups causes starch granule structure to weaken, which increases the solubility. Thus, in general, starch solubility increases after oxidation. In this case, the carboxyl groups strongly affect the amylose degradation.

Meanwhile, amylose molecules that degraded into short-chain molecules begin to dissolve in water, which increases the solubility of starch in water [33]. The solubility of starch is influenced by the amylose content that is released from the starch chain [52]. The carboxyl group has a more significant electronegative charge than the hydroxyl group. Because a carboxyl group causes electrostatic repulsion between molecules, it is responsible for interfering with their intermolecular association, which further increases their solubility [20,53].

The swelling power of modified starch, or its ability to expand, is a property that characterizes the development of material. The contact between the starch granules and water causes the granule bodies to swell; thus, the swelling power indicates the ability of starch to hydrate under different conditions [21]. The swelling power of starch occurs because of the increased volume during baking and the maximum weight of the starch in water [54] or because of the non-covalent bonds between starch molecules at the amorphous (irregular) region of starch [55]. Weakened hydrogen bonds during the oxidation process cause the hydration of starch granules. The hydrophilic character of the starch granules, which can bind the hydrogen in water molecules to absorb water, cause the increase in swelling power [56]. Several factors that can influence the capacity of water to bind to starch granules include the molecular weight and ratio of amylose-amylopectin, the distribution of their molecular weights, degree of branching, and the length of the branch of the amylopectin molecule [57].

Overall, the value of swelling power decreases with an increasing oxidation time. The structural disintegration in starch granules during the modification process reduces swelling [21]. Amylose, which has a linear structure, is more easily depolymerized than amylopectin during oxidation; native corn starch was lower than oxidized corn starch at 95 °C [58]. The hydrolysis of the amylopectin chain at high temperatures causes this phenomenon; the formation of pores in the granules increases water absorption during heating, but the pores are weak in retaining the absorbed water centrifugation. A similar reduction of swelling power that was based on the oxidation process was reported for mucuna beans [59]. Additionally, the swelling power of modified sago starch decreased with the increase of oxidation time, and the swelling power of the native sago starch was found to be higher than the modified sago starch [29].

Here, oxidation did not significantly change the morphology of sago starch. Some of the sago starch granules underwent erosion during oxidation, and the surface became rough, with several cracks and pores appearing on the surface, while the edges partially disappeared into irregular shapes (Figure 13b–f). However, such change occurred in a few granules that became turned; modified starch granules were mostly shaped like native granules with a smooth surface. The damage on the starch granule only occurred in the outer structure of the granules and in small areas, because most of the oxidation occurred on the surface of the granule. Thus, the surface of starch molecules is more accessible to oxidize than its crystalline region due to its higher accessibility.

According to Klein et al., pH greatly affects the surface morphology of starch granules [31]. Oxidation changes the surface and shape of the potato starch granules in an aqueous solution; however, the effect of oxidation is difficult to detect on corn starch granules. Similar observations were made in ozone-oxidized potatoes and corn while using SEM [60]. The native starch presented a regular and smooth surface, while, after ozonation, their surface becomes fibrous and rough, especially on potato starch granules. Similar results were obtained in potato starch with high-pressure treatment [61] and modification with octenyl succinic anhydride [16]. Zhou et al. studied native and modified potato starch with sodium hypochlorite processing [51]; they observed that the weakening of the starch’s crystal structure caused the starch granules to crack at higher oxidant concentrations. Other research found that, after oxidation, potato starch granules became rougher and more heterogeneous with an increasing processing time [20].

The O–H group of sago starch with a starch concentration of 20% and pH 10 had a higher value than the native starch. The increase of the percentage of transmittance demonstrates the weakening of the O–H bond in sago starch, which indicates the oxidation of O–H bonds to the carbonyl and carboxyl groups [7,43]. Additionally, the C–H bonds in modified sago starch show an increase in the percentage of transmittance, which suggests a weakening of the hydrogen bonds. Additionally, in the C=O bond, the rate of transmittance in modified sago starch was increased when compared to the native starch, indicating a weakening of the C=O bond. Sago starch at 20% and pH 10 experienced a strengthening of the C-N bond, as indicated by the change in the percentage of transmittance. The Maillard reaction causes the C-N bond when the carbonyl group (C=O) binds to the protein in starch [62].

These results (Table 1) indicate that endothermic peaks occur in native starch (106.08 ± 2.95 °C) or oxidized starch (in the range of 103.18 ± 2.01 °C–116.93 ± 3.01 °C) [47]. The combination of 10% concentration and pH 4 had lower To, Tp, and Tc values when compared to native starch, while the variable with 30% concentration had opposite conditions, even with the same pH. This result is linear with the enthalpy value, where the 30% concentration has an enthalpy greater than the original starch enthalpy and 10% concentration. The increase in starch concentration coincided with an increase in To, Tp, and Tc [63]. The values of To, Tp, and Tc presented in this study have the same results as the research that was conducted by Boonna & Tongta (2018), which examined the thermal properties of cassava starch after going through a modification process using the annealing method, heat moisture treatment, where the modification increases the thermal stability when compared to native cassava flour [64]. The weakened intermolecular bonds are related to the thermodynamic characteristics of starch. The carboxyl groups in the starch granules affect the characteristic amylopectin crystal structure [65]. In addition, the chemical modification of starch granules can reflect the surface appearance, fractures, and pores, and facilitate water penetration in the granules [66]. The thermal properties of starches from varied sources are due to the starch composition, ratio of amylose-amylopectin, residual lipids and protein, morphology, molecular structure, and the starch granules distribution [21].

## 5. Conclusions

The modification of sago starch was found to affect physicochemical properties. Oxidation with ozone significantly affected the solubility, swelling power, and carbonyl and carboxyl content of the native starch. Native starch is more oval-shaped and irregularly shaped than modified starch, according to SEM analysis. Additionally, in the FTIR analysis results, between native starch and modified starch changes in absorption at specific wavelengths, the spectral patterns of all the samples were similar. In addition, the oxidized sago starch showed identical XRD patterns to that of native starch. On the other hand, DSC analysis revealed that the profile of gelatinization and retrogradation temperatures of oxidized starches was insignificantly different from native starch.

## Figures and Tables

**Table 1:** Thermal properties of native and ozone-oxidized sago starches.

Sago Starch	Variables	To (°C)	Tp (°C)	Tc (°C)	ΔH (J/g)
Ozone-oxidized sago starch	10% pH 4 25 min	67.53 ± 2.05 ^a^	103.18 ± 2.01 ^a^	151.94 ± 2.45 ^a^	−280.24 ± 3.01 ^d^
Ozone-oxidized sago starch	20% pH 4 25 min	83.87 ± 1.68 ^bc^	112.19 ± 3.35 ^c^	158.43 ± 3.25 ^b^	−373.64 ± 2.76 ^a^
Ozone-oxidized sago starch	20% pH 7 25 min	78.97 ± 1.58 ^b^	113.71 ± 2.31 ^c^	150.12 ± 1.46 ^a^	−363.02 ± 5.02 ^b^
Ozone-oxidized sago starch	20% pH 10 25 min	80.86 ± 1.60 ^b^	106.31 ± 3.54 ^b^	158.34 ± 3.05 ^b^	−298.18 ± 2.50 ^d^
Ozone-oxidized sago starch	30% pH 4 25 min	88.23 ± 1.76 ^c^	116.93 ± 3.01 ^d^	157.66 ± 3.21	−361.39 ± 5.23 ^b^
Native sago starch	No variable	80.98 ± 1.32 ^b^	106.08 ± 2.95 ^b^	151.61 ± 2.92 ^a^	−310.34 ± 4.07 ^c^

Different letter superscripts within the same columns present statistically significant different at *p* < 0.05.

## Data Availability

The datasets generated during and/or analyzed during the current study are available from the corresponding author on reasonable request.
